# Exploring the Acute Effects of the Daily Mile™ vs. Shuttle Runs on Children’s Cognitive and Affective Responses

**DOI:** 10.3390/sports10100142

**Published:** 2022-09-22

**Authors:** Ricardo M. G. Martins, Michael J. Duncan, Cain C. T. Clark, Emma L. J. Eyre

**Affiliations:** Centre for Sport, Exercise and Life Sciences, Coventry University, Coventry CV1 5FB, UK

**Keywords:** physical activity, cognitive performance, inhibition, children, executive function, affective responses

## Abstract

Background: This study investigated the acute effects of two physical activity (PA) bouts on children’s cognitive and affective responses. Methods: Twenty-nine participants (16 boys and 13 girls; M_age_ = 9.34 years, SD = 0.48), using a within-subjects crossover design, performed three 15-min conditions: (a) TDM—The Daily Mile™; (b) 12 repeated 30–45-s shuttle runs at ≥ 85% HR_MAX_; and (c) a sedentary control condition. Cognitive performance (i.e., Stroop, Digit Span, and Corsi blocks) was measured before PA and 1 and 30 min post-PA. Felt Arousal and Feeling Scale self-report scales were administered before, during, and after PA. Results: The results show no changes following the TDM condition relative to the sedentary control condition in cognitive responses. However, when comparing the shuttle runs condition to the sedentary control condition, participants showed higher arousal, an improved reaction time, and lower self-reported pleasure at 1 min post-PA. Nevertheless, at 30 min post-PA, participants’ pleasure values were higher in the shuttle runs condition than they were before PA. Conclusions: When comparing PA conditions, shuttle runs enhanced reaction time and might thus be seen as an option to implement or modify PA opportunities in school settings.

## 1. Introduction

There has been a growing interest in the relationship between physical activity (PA) and executive functioning (EF), an aspect of cognitive capability. EF is usually categorised into three main sub-domains: inhibition, cognitive flexibility, and working memory [1], and these domains have been positively associated with academic performance [2,3]. Seeking ways to improve EF in school-aged children, investigators have linked short-duration PA of about 10–20 min to improved EF from 1 to 30 min post-PA [4,5]. However, the association between PA and EF depends on various factors such as PA intensity, duration, and type, as well as the time of assessment and the research participant’s age, sex, and body mass index [6]. Therefore, the optimal PA dose for enhancing EF in 6–12-year-old children is still unknown.

Regarding the PA dose, Chang et al. [7] concluded from a meta-analysis of studies of the acute PA effects on EF across participants aged 5–60+ years that low- to moderate-intensity PA bouts (i.e., 50–76% HR_MAX_) were associated with immediate EF benefits, while high-intensity PA (i.e., 77–93% HR_MAX_) was more effective at 1 min post-PA. In a small number of EF studies in children that examined intensity variance, low to moderate effect sizes for immediate EF gains from both low and high intensities were shown [5,8,9,10]. Considering PA duration, Chang et al. [7], Williams et al. [5], and Ai et al. [4] reported that an acute PA bout, or a single bout of PA, of more than 10 min elicited higher EF for both young children and adults. However, other researchers have shown that additional relevant variables include the type of research design, the type of PA employed, the type and timing of EF assessment tools, and the participant’s age and physical fitness [6]. Relatively few studies have focused on children, making it difficult to ascertain a specific PA intensity and duration for eliciting improved EF responses among children. In studies of children, positive EF effects have been more consistently observed at 20–30 min post-PA [10,11,12,13,14,15,16,17], but less is known of how long such benefits might last. These studies have used different durations of PA protocols, varied EF assessment tools, and different real-world environments. These studies also varied in the degree to which participants adhered to or enjoyed and were engaged in the PA.

In this context, and to examine the acute impact on children’s EF of varied types of running in school settings, while also tracking children’s affective responses and perceived exertion, this study compared The Daily Mile™—a self-paced PA—intermittent shuttle runs, and a sedentary control condition. The Daily Mile™ consists of running or jogging at a self-selected pace for approximately 15 min outside the school building. Among studies investigating the acute effects of The Daily Mile™ on EF performance, Morris et al. [18] found no significant math fluency improvement compared to a control condition, while Booth et al. [19] found that, compared to a near-maximal exhaustion activity, i.e., a 20-m bleep test, The Daily Mile™ was associated with better response inhibition and verbal memory at post-PA testing within a 20-min interval. However, Booth et al. [19] relied on participants’ self-collected data, and the EF assessment timing was imprecise. More recently, Hatch et al. [20] found no effects on inhibitory responses, visual working memory, and cognitive flexibility following TDM compared to a rest control post-PA. Additionally, the authors explored factors of enjoyment and concluded that TDM induced mixed perceptions in children as some participants seemed to enjoy it due to its outdoor location, social context, and self-paced nature while others found it boring due to its repetitiveness [20]. Given this, alternative ways to promote PA in school settings or modify TDM to achieve the benefits of being active and simultaneously improve EF are needed.

Since children’s daily PA patterns are generally sporadic, spontaneous bouts of high-intensity activity may better mirror their natural intermittent PA [21]. This form of PA may be easily undertaken in school settings. To our knowledge, seven studies of EF effects from PA used running protocols [11,15,16,19,22,23,24]. These studies varied in PA intensities and the type and duration of EF assessments, and the results have led to no clear consensus. More specifically, only two studies investigated the EF effects of continuous versus intermittent running. Lambrick et al. [15], using moderate-intensity 15-min runs, found improved reaction times at 1–30 min post-PA from both running conditions, but reaction time benefits were larger for the intermittent condition. Comparing self-paced versus high-intensity beep-cued runs, Booth et al. [19] found enhanced inhibition and verbal memory only from the self-paced runs, leading to a situation where more studies are needed to examine the effects of different running PA bouts in school-aged children’s EF.

The present study aimed to compare participants’ response inhibition and visual and verbal recall following (a) The Daily Mile™, (b) high-intensity intermittent shuttle runs, and (c) a sedentary control condition. To better characterise participants’ affective responses and the likelihood of adherence to these activities, self-report data regarding participants’ affect, ratings of perceived exertion, and arousal were also collected.

## 2. Materials and Methods

### 2.1. Study Design and Participants

Twenty-nine participants (16 boys and 13 girls; M_age_ = 9.34 ± 0.48 years) were recruited (convenience sampling) from two primary schools in West Midlands, UK. Following institutional ethical approval (P91970), informed parental consent and child assent were obtained, and a power calculation was employed to determine the minimum number of participants required [24] for a mixed ANOVA; the parameters were set as follows: Power = 0.8, α = 0.05, and ES(f) = 0.14–0.25 or η^2^p = 0.02–0.06. One participant (i.e., 1 out of 30) was excluded from the study for failing to complete both assessment trials, and any participants with any contraindication to PA engagement (e.g., musculoskeletal injury, taking medication, cognitive impairments, colour blindness, and/or cardiovascular problems) or with any mental health disorders/cognitive impairments, as reported by the classroom teacher and/or through a physical activity readiness questionnaire, were excluded. Data from boys and girls were combined together in line with previous studies following similar protocols [12,15].

### 2.2. Anthropometric Measures

Participants’ body mass, height, and sitting height were measured, and Mirwald et al.’s [25] equations were used to calculate participants’ peak height velocity (PHV). Maturity offset was defined by the difference in years between the PHV age and chronological age. For each anthropometric measure, two measurements were obtained. However, if the difference between measurements was larger than 4 mm for either height or sitting height and 0.4 kg for weight, a third measurement was taken, and the median value was used [26]. Participants’ BMI was calculated with the following formula: body mass (kg) divided by stature (m) squared, and the determined age- and sex-specific cut-off points of Cole et al. were used [27].

### 2.3. Procedures

A research randomiser (https://www.randomizer.org, accessed on 14 April 2019) was used to assign each participant to one of the three PA conditions: (a) The Daily Mile™, (b) shuttle runs, and (c) the sedentary control. In the sedentary control condition, participants sat and watched a video (One Breath Around The World—YouTube, 2019) with minimal or no mental engagement for the same duration as the two running conditions [28].

The first author collected all participant anthropometric data in the morning before participants engaged in the PA or sedentary control conditions. Participants from the two schools performed the cognitive tests in a quiet area of the school. To standardise and attempt to avoid learning or practice effects on the cognitive measures between measurements, a test familiarisation session was employed on the first day. All participants performed at least 3 to 4 full trials of each cognitive test, as prior research has shown that learning effects can be diminished after 2–4 test administrations [29,30,31]. A washout period of 48–72 h between all conditions was implemented [15], and all participants were asked to be well-rested and hydrated without engaging in any vigorous PA/exercise or PE lessons the previous day. If any participants reported being tired, not in the mood, or were stressed or visibly disturbed on any day, they were allowed to run on a different day.

Following 10 min of seated rest, the participant’s resting heart rate (Polar FT1 Heart Rate Monitor, Polar Electro, Finland) was obtained. HR_MAX_ was predicted using the 208–0.7 (age) equations [32], as these equations can closely predict HR_MAX_ for children aged 7–17 years [33]. On a different day, at the same time of day, to examine participants’ level of physical fitness, they engaged in a 550-m walk/run. Level of fitness has been found to potentially impact cognitive outcomes, and this 550-m walk/run test has been previously validated for 8–13-year-old children [34]. These values are reported in Table 1.

### 2.4. PA Protocol

**Figure 1:**
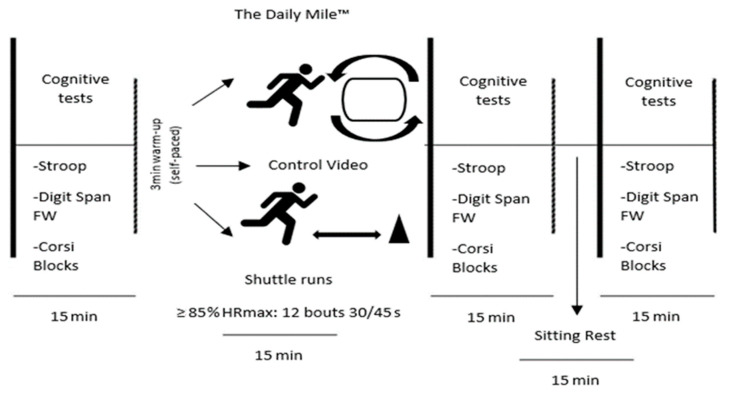
Time course and procedures for TDM, control, and shuttle run conditions.

All participants completed the sedentary control and the two PA conditions in the school playgrounds. The shuttle runs consisted of 12 bouts of 30-s runs at ≥ 85% HR_MAX_ with resting periods between them of 45 s (slow walk/rest). This protocol followed previous studies using intermittent and high-intensity PA with intensities between 50 and 90% HR_MAX_ [24,35]. In the TDM condition, the participants performed this self-paced PA following the guidelines provided to schools (The Daily Mile Foundation, 2019). The two PA protocols and the sedentary control activity were of 15-min durations. Participants doing shuttle runs were informed of their 85% HR_MAX_, and during familiarisation, they were instructed on how to maintain the expected shuttle run pace. A description of the protocol is presented in Figure 1. The same researcher monitored the participants’ HR. Participants also completed self-report scales measuring arousal, affect, and ratings of perceived exertion (RPEs) each time they underwent cognitive assessments.

### 2.5. Assessment of Inhibitory Responses and Verbal and Visual Recall

The open-source software resource PEBL (The Psychology Experiment Building Language, version 2.1, MI, USA) [36] was used to administer the Corsi block test (as adapted by Corsi [37] and Kessels et al. [38]), the Digit Span Forwards subtest (adapted from Wechsler [39]), and the Stroop test (adapted from Stroop [40]). Cognitive tests were administered at 1 and 30 min post-PA, using standard instructions provided by the PEBL software [36]. Any participants with visual limitations were asked to wear their glasses or contact lenses. The cognitive tests were administered in the following fixed order: Stroop test, Digit Span, and Corsi blocks; this test battery averaged 10–15 min in duration.

#### 2.5.1. Stroop Colour–Word Test

The Stroop test measures response inhibition (cognitive control and reaction time) mediated by prefrontal cortical functioning [41], and it has been widely used to assess the effects of acute PA on cognitive inhibition [7,15,42,43]. The Stroop test has good reliability (*r* > 0.80) and validity for children (5–17 years of age) [44]. The Stroop test includes a cognitive interference task and consists of presentations of words printed in four different colours (blue, yellow, green, and red). Participants were first asked to name the colours as quickly and accurately as possible, responding by clicking on the respective keyboard button (1, 2, 3, and 4). All participants were then presented with a series of trials in which they saw 30 words printed in different ink colours. In each trial, the colour word meanings might have been congruent with the ink colours in which they were printed (e.g., the word green written in green ink), or they might have been incongruent (e.g., the word green written in blue ink), requiring participants to utilise cognitive control to inhibit an initial response and identify the ink colour rather than the word meaning and accurately read the colour word or neutral word (written in black ink). The Stroop interference score considered the response times for incongruent responses in relation to congruent responses (i.e., reaction times for incongruent minus congruent stimuli). Participant and practice requirements, stimulus fixation times, and measures of performance were software standards [36] and followed the same protocol as previous studies [43,45].

#### 2.5.2. Digit Span

The Digit Span (DS) test is a verbal recall test adapted from the Wechsler intelligence scales [39]. This test consisted of an increasingly long number sequence to recall, with the numbers of the sequences presented at a one-second rate over two trials beginning with a 2-digit sequence. The test ended with recall failures on both trials of a given sequence length or all trials administered, including a maximal sequence length of 10 digits. Within the standardised administrative guidelines of the PEBL software, test measures were the total number of 2-trial sequences correctly recalled and the length of the last correctly recalled memory span. This test has shown moderately high reliability (0.83–0.89) [46,47], and computerised versions assure standardised test administration [48,49].

#### 2.5.3. Corsi Block Test

The Corsi block test is a visual recall test based on work by Corsi [37] and Kessels et al. [38]. In a computer screen presentation of this task, participants must recall the specific order of blinking blocks from within a fixed array of nine blocks. The block images blinked one by one at inter-stimulus intervals of 1000 ms. The number of blinking blocks in the sequence increased in length, beginning with three. To measure participants’ performance, scores of the number of correct sequences remembered (i.e., the block span), the total score (i.e., the span multiplied by the total correct), the total number of correct trials, and memory span (minimum list length achieved or the total recalled correctly divided by the number of lists of each length) were obtained.

### 2.6. Arousal, Affect, and Effort Scales (FAS, FS, and RPE)

Participants self-reported arousal on the Felt Arousal Scale (FAS) [50], affect on the Feeling Scale (FS) [51], and perceived physical effort or rating of perceived exertion (RPE) on the 1–10 pictorial Children’s OMNI scale [52]. Details regarding each of these scales are presented below (please see Section 2.6.1). Standardised instructions on using these scales were provided, and participants were given time to practice them during the familiarisation procedure. The same researcher administered all the scales at three time periods: before PA, during PA (coded as mid-trial 1 and mid-trial 2, describing the median values during the first and second halves of the bout, respectively), and at 1 and 30 min post-PA. Central measurements (medians) were used for data analysis.

#### 2.6.1. FS and FAS Scales

Participant arousal was measured with the single-item FAS [50] consisting of a 6-point scale, with anchors at 1 for low arousal (e.g., relaxation, boredom, or calmness) and 6 for high arousal (e.g., excitement, anxiety, or anger). Participant affective valence (pleasure/displeasure) was measured with the FS [51], on which participants rated their various current feelings on an 11-point bipolar scale ranging from +5 to −5, with verbal anchors of *very good* (+5), *good* (+3), *fairly good* (+1), *neutral* (0), *fairly bad* (−1), *bad* (−3), and *very bad* (−5). Following data collection, a circumplex model was used [53] to visually represent participants’ reported arousal and feelings [54]. The FS and FAS were represented in four quadrants of a 2X2 arousal/feelings grid consisting of (a) unaroused/pleasant affect (e.g., relaxation and pleasure), (b) unaroused/unpleasant affect (e.g., relaxation and displeasure), (c) aroused/unpleasant affect (e.g., excitement and displeasure), or (d) aroused/pleasant affect (e.g., excitement and pleasure). The FS and FAS have shown moderate reliability, with correlations ranging from 0.41 to 0.59 and 0.47 to 0.65, respectively, when used within the Affect Grid [55], and these measures have shown good convergent validity with similar established measures [56].

#### 2.6.2. RPE

The 1–10 pictorial Children’s OMNI rating of perceived exertion (RPE) scale [52] has a range of numbers familiar to youth (1 to 10) and uses age-appropriate verbal expressions as descriptors of effort during PA, with rating anchors that range from ‘*not tired at all’* (0) to ‘*very, very tired*’ (10). This instrument has been previously validated for children between 6 and 13 years old, with significant correlations between RPE and %$\dot{V}$O^2^_MAX_ (*r* = 0.41–0.60, *p* < 0.001) and RPE and HR (*r* = 0.26–0.52, *p* < 0.01) [52].

### 2.7. Statistical Procedures

Cognitive measures were analysed using the Statistical Package for the Social Sciences (SPSS v.27.0, IBM Inc., New York, NY, USA). A series of three (conditions: The Daily Mile™, shuttle runs, and sedentary control) by three (times: before PA, 1 min post-PA, and 30 min post-PA) mixed analyses of variance (ANOVAs) was conducted. All cognitive data distributions were tested for normality with histograms and Q–Q values, and the values were shown to be within the recommended range plots for skewness (2,+2) and kurtosis (7,+7) [57,58]. Where sphericity assumptions were violated, Greenhouse–Geisser corrections were used to adjust the degrees of freedom, and these are reported. For significant effects (*p* < 0.05), follow-up post hoc tests using LSD were used as the criteria of K = 3 groups or time points were met [59], and the magnitude of mean differences was interpreted using partial eta-squared, η^2^p, effect size descriptions as follows: 0.01 (small), 0.06 (medium), and 0.14 (large) [60].

As the affect and RPE scales did not meet the normality criteria [57,58], a non-parametric repeated measures ANOVA (Friedman) using Jamovi (V.1.2.17) was employed. Pairwise comparisons with Durbin–Conover equations [61] were used to discern differences across the three time points (before PA, mid-trial 1 and 2, and 1 min and 30 min post-PA). The results were reported using medians (MED) and interquartile ranges (IQR).

## 3. Results

### 3.1. Strop Test, Digit Span, and Corsi Blocks Outcomes

**Figure 2:**
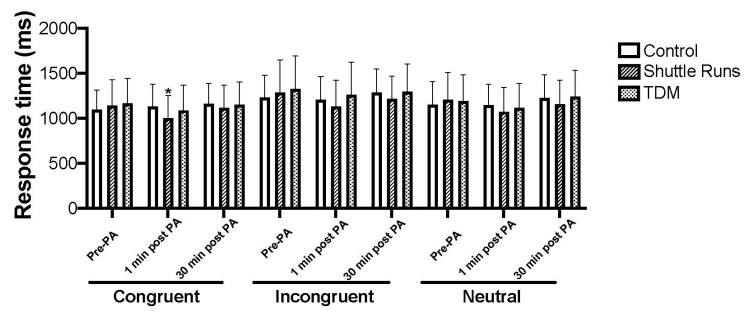
Stroop test reaction time score for the control, shuttle runs, and TDM conditions (Ms and SDs). * Significant at *p* < 0.05 (compared to control condition).

The participants’ descriptive statistics and cognitive outcomes are displayed in Table 2 and Figure 2. There were no significant changes in the participants’ accuracy for these stimuli (all *p* > 0.05), and there was no significant effect of session order, gender, PHV, BMI, and fitness scores (all *p* > 0.05).

#### 3.1.1. Stroop Test

There was a statistically significant interaction effect of PA condition on assessment time for Stroop congruency with a medium effect size (F_(4, 92)_ = 3.3, *p* = 0.013, η^2^p = 0.127), and there was a main effect of assessment time with a large effect size (F_(2, 46)_ = 3.7, *p* = 0.033, η^2^p = 0.137) (before PA vs. 1 min post-PA: *p* = 0.012, M difference = 61 ms, SD = 22; 1 min post-PA vs. 30 min post PA: *p* = 0.031, M difference = 75 ms, SD = 33). Post hoc analyses revealed significantly quicker reaction times on congruent stimuli in the shuttle runs vs. the control condition at 1 min post-PA (M difference = 119 ms, SD = 37, *p* = 0.004; Table 2); there were no statistically significant changes between the TDM and control conditions (all *p* > 0.05; Table 2). For incongruent Stroop stimuli, there only was a main effect of time, with a large effect size (F(1, 30) = 5, *p* = 0.024, η^2^p = 0.179) (before PA vs. 1 min post-PA: *p* = 0.001, M difference = 81 ms, SD = 22; 1 min post-PA vs. 30 min post-PA: *p* = 0.004, M difference = −68 ms, SD = 21). For neutral stimuli, there was a statistically significant interaction effect of condition by time with a medium effect size (F(4, 92) = 2.5, *p* = 0.049, η^2^p = 0.097) and a significant main effect of time (F_(2, 46)_ = 6.7, *p* = 0.004, η^2^p = 0.217) with a large effect size (before PA vs. 1 min post-PA: *p* = 0.022, M difference = 70 ms, SD = 28; 1 min post-PA vs. 30 min post-PA: *p* = 0.001, M difference = −95 ms, SD = 24). Post hoc analyses showed no differences between the control and experimental conditions for incongruent and neutral stimuli (all *p* > 0.05; Table 2). However, within conditions, participants improved in the shuttle runs condition at 1 min post-PA compared to before PA (*p* = 0.005, M difference = 131 ms, SD = 42; Table 2). On the other hand, comparing 1 min post-PA to 30 min post-PA, there was a significantly slower reaction time at 30 min post-PA (*p* = 0.028, M difference = 78 ms, SD = 33; Table 2). Similar to the shuttle runs condition, in the TDM condition, there was a significantly slower reaction time at 30 min post-PA compared to 1 min post-PA (*p* = 0.007, M difference = 126 ms, SD = 42; Table 2). However, when comparing condition effects at 30 min post-PA, the shuttle runs condition was associated with significantly quicker reaction times than the TDM condition (*p* = 0.050, M difference = 85 ms, SD = 41; Table 2). Stroop interference scores did not change significantly for the condition by time interaction, or for main effects of time and condition. There were no significant time, condition, or interaction effect changes in participants’ accuracy over the assessment times for any of the Stroop stimuli (all *p* > 0.05, Table 2).

#### 3.1.2. Digit Span

There were no significant participant verbal recall effects for the condition by time interaction or for time or condition main effects (all *p* > 0.05; Table 2).

#### 3.1.3. Corsi Blocks

In the visual recall test, there were no significant changes in participants’ performance for the condition by time interaction or for the main effects of time or condition (all *p* > 0.05; Table 2). More specifically, the non-significant interaction effect (condition by time) for correct trials (F(4, 92) = 1.2, *p* = 0.3, η^2^p = 0.048) showed a small to medium effect size, and the interaction effect for memory span (F(4, 92) = 1.3, *p* = 0.287, η^2^p = 0.052) showed a medium effect size. The non-significant interaction effect on block span revealed a small effect size (F(4, 92) = 0.5, *p* = 0.561, η^2^p = 0.032).

**Table 2:** Scores for the Stroop test, Digit Span test, and Corsi test for the control, shuttle runs, and TDM conditions (Ms and SDs).

	Control			Shuttle Runs			TDM		
	Pre-PA	1 min	30 min	Pre-PA	1 min	30 min	Pre-PA	1 min	30 min
**Stroop test**									
**Congruent**	1097 (216)	1130 (249)	1158 (228)	1118 (296)	*●991 (257)	●1086 (255)	1155 (279)	●1072 (272)	1125 (248)
Boys Girls	1042 (185) 1150 (237)	1052 (202) 1203 (273)	1145 (255) 1171 (209)	1036 (262) 1219 (310)	936 (210) 1059 (286)	1033 (208) 1153 (297)	1098 (270) 1232 (283)	1024 (232) 1136 (318)	1075 (231) 1192 (265)
**Accuracy**	0.90 (0.1)	0.92 (0.1)	0.89 (0.1)	0.94 (0.1)	0.93 (0.1)	0.91 (0.1)	0.93 (0.1)	0.91 (0.1)	0.93 (0.1)
Boys Girls	0.90 (0.1) 0.90 (0.1)	0.90 (0.1) 0.93 (0.1)	0.88 (0.1) 0.92 (0.1)	0.95 (0.1) 0.94 (0.1)	0.93 (0.1) 0.94 (0.1)	0.90 (0.1) 0.93 (0.1)	0.92 (0.1) 0.93 (0.1)	0.88 (0.1) 0.95 (0.1)	0.91 (0.1) 0.94 (0.1)
**Incongruent**	1231 (246)	1207 (255)	1284 (263)	1269 (364)	1109 (282)	1186 (254)	1306 (359)	1234 (347)	1272 (305)
Boys Girls	1116 (161) 1337 (268)	1166 (246) 1245 (267)	1225 (259) 1338 (265)	1181 (276) 1378 (437)	1007 (211) 1235 (314)	1117 (217) 1271 (279)	1122 (305) 1417 (407)	1165 (241) 1327 (447)	1199 (228) 1370 (373)
**Accuracy**	0.89 (0.1)	0.90 (0.1)	0.86 (0.1)	0.90 (0.1)	0.93 (0.1)	0.90 (0.1)	0.88 (0.1)	0.89 (0.1)	0.88(0.1)
Boys Girls	0.87 (0.2) 0.92 (0.1)	0.88 (0.1) 0.92 (0.1)	0.85 (0.1) 0.88 (0.1)	0.90 (0.1) 0.90 (0.1)	0.91 (0.1) 0.94 (0.1)	0.94 (0.1) 0.84 (0.1)	0.90 (0.1) 0.86 (0.1)	0.91 (0.1) 0.88 (0.1)	0.89 (0.1) 0.87 (0.1)
**Neutral**	1149 (257)	1146 (233)	1228 (257)	1185 (307)	●1059 (262)	●1128 (264)	1178 (285)	●1108 (261)	●1213 (285)
Boys Girls	1138 (291) 1160 (233)	1129 (243) 1162 (232)	1178 (265) 1274 (260)	1013 (255) 1282 (315)	1013 (255) 1116 (269)	1087 (215) 1181 (316)	1114 (237) 1264 (330)	1069 (214) 1159 (316)	1144 (261) 1305 (300)
**Accuracy**	0.90 (0.1)	0.92 (0.1)	0.87 (0.1)	0.92 (0.1)	0.94 (0.1)	0.92 (0.1)	0.92 (0.1)	0.92 (0.1)	0.91 (0.1)
Boys Girls	0.87 (0.1) 0.93 (0.1)	0.89 (0.1) 0.94 (0.1)	0.86 (0.1) 0.88 (0.1)	0.92 (0.1) 0.92 (0.1)	0.93 (0.1) 0.95 (0.1)	0.91 (0.1) 0.94 (0.1)	0.92 (0.1) 0.93 (1)	0.90 (0.1) 0.94 (0.1)	0.90 (0.1) 0.92 (0.1)
**Digit Span**									
**Correct sequences**	5.2 (2.4)	5.2 (2.4)	5.1 (2.3)	4.9 (1.7)	5.3 (1.9)	5.2 (1.6)	5.0 (1.9)	5.5 (2.2)	5.4 (2.1)
Boys Girls	4.7 (2.1) 5.7 (2.6)	4.8 (1.8) 5.7 (2.8)	4.7 (2.2) 5.4 (2.5)	4.3 (1.7) 5.6 (1.4)	4.5 (1.6) 6.3 (1.7)	5.2 (1.8) 5.2 (1.5)	4.8 (2.0) 5.3 (1.9)	5.6 (2.3) 5.4 (2.0)	5.2 (2.4) 5.8 (1.7)
Memory span	5 (1.2)	5.1 (1.4)	4.9 (1.4)	4.8 (1)	5.2 (1)	5.2 (1)	5.1 (1.2)	5.4 (1.3)	5.2 (1.2)
Boys Girls	4.7 (1.1) 5.2 (1.4)	4.9 (1.1) 5.3 (1.7)	4.6 (1.3) 5.7 (2.6)	4.3 (1) 5.2 (1)	4.8 (1) 5.8 (1)	5.2 (1) 5.1 (1)	4.8 (1.2) 5.4 (1.2)	5.4 (1.5) 5.3 (1.1)	5.0 (1.4) 5.5 (1.0)
**Corsi**									
Block span	5.1 (1)	5 (1.1)	4.6 (1.3)	4.9 (1.1)	4.9 (1.1)	4.7 (0.8)	4.9 (1.1)	4.8 (1.0)	4.8 (0.8)
Boys Girls	5.3 (1) 5 (1)	4.9 (1.4) 5 (1)	4.5 (1.6) 4.6 (1)	5.1 (1.2) 4.5 (0.78)	5.1 (1.1) 4.7 (1)	4.8 (1) 4.6 (1)	4.9 (1.4) 4.8 (0.6)	4.9 (1.1) 4.8 (1.0)	4.9 (0.8) 4.8 (0.8)
Correct trials	6.8 (1.7)	6.5 (2)	5.9 (2)	6.4 (1.7)	6.7 (1.8)	6.5 (1.7)	6.6 (1.8)	6.5 (1.7)	6.4 (1.7)
Boys Girls	6.7 (1.9) 6.9 (1.6)	6.6 (2.3) 6.5 (1.8)	5.9 (2) 5.9 (2)	6.9 (1.7) 5.6 (1.5)	7.1 (1.9) 6.2 (1.6)	6.8 (1.8) 6.2 (1.5)	6.7 (2.1) 6.3 (1.5)	6.7 (1.9) 6.2 (1.3)	6.6 (1.7) 6.3 (1.7)
Memory span	4.4 (0.8)	4.3 (1)	4 (1)	4.2 (0.8)	4.3 (0.9)	4.2 (0.8)	4.3 (1)	4.2 (0.8)	4.1 (0.8)
Boys Girls	4.4 (1) 4.5 (1)	4.3 (1.1) 4.3 (1)	4 (1) 4 (1)	4.5 (0.8) 3.9 (0.8)	4.4 (0.9) 4 (0.8)	4.4 (0.9) 4.0 (0.8)	4.3 (1.1) 4.2 (0.7)	4.4 (1.0) 4.1 (0.7)	4.3 (0.8) 4.1 (0.8)

Represented by milliseconds (ms), accuracy (range 0–1), and memory span. * Significant at *p* < 0.05 (compared to control condition). ● Significant at *p* < 0.05 (within conditions compared to before).

### 3.2. Affect Scales

Participants’ descriptive statistics and cognitive outcomes are displayed in Table 2. There were no significant changes in the participants’ accuracy for these stimuli (all *p* > 0.05), and there was no significant session order effect (all *p* > 0.05).

#### 3.2.1. Feeling Scale

**Figure 3:**
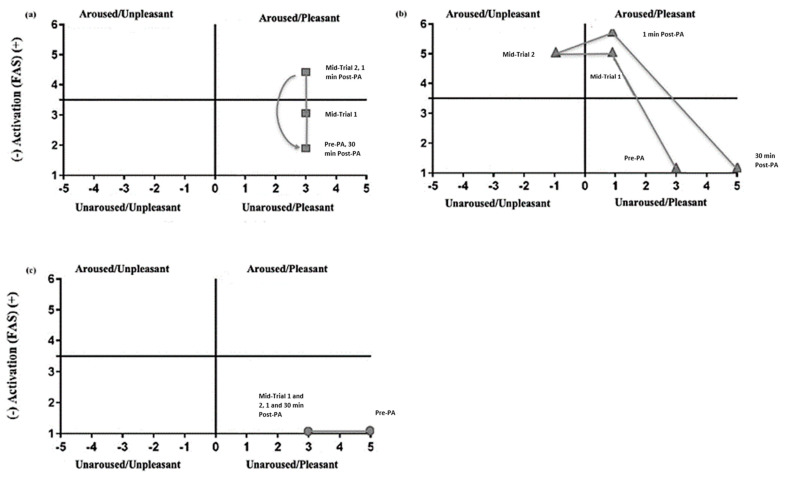
FS and FAS as analysed using the circumplex model by condition: (**a**) TDM, (**b**) shuttle runs, and (**c**) control.

Statistically significant differences were reported for the FS across the conditions (χ^2^(14) = 102, *p* = 0.001). In the control condition, there were statistically significant differences across the different time points; participants reported higher values (pleasure) before PA compared to the mid-trial 1, mid-trial 2, and 1 min post-PA time points (all *p* < 0.05; Table 3). There were significantly lower values of pleasure reported in both experimental conditions at mid-trial 1, mid-trial 2, and 1 min post-PA (all *p* < 0.05; Table 3). Across conditions, there were significantly lower values of pleasure at the before PA time point for the shuttle runs condition than in the other conditions. Nevertheless, across the time points in the shuttle runs condition, there were significantly higher values of pleasure at 30 min post-PA compared to before PA (all *p* < 0.05; Table 3). Comparing both running conditions, there were high values of displeasure in the shuttle runs condition at mid-trial 2 and 1 min post-PA compared to the TDM condition. The changes between the FS and FAS scores for all conditions are illustrated and can be observed in the circumplex model [53] (Figure 3).

#### 3.2.2. Felt Arousal Scale

Statistically significant differences were found for FAS scores across the conditions (χ^2^(14) = 185, *p* = 0.001). There were no significant changes in the control condition across the different time points (all *p* > 0.05; Table 3), and there were increased arousal levels in both running conditions after the beginning of the PA bout at mid-trial 1, mid-trial 2, and 1 min post-PA when compared to the control condition and the before PA time point (all *p* = 0.001; Table 3). There were significantly higher arousal values in the shuttle runs condition compared to the TDM condition (all *p* < 0.05; Table 3).

#### 3.2.3. RPE

Statistically significant differences were found for the OMNI scale across the conditions (χ^2^(14) = 265, *p* = 0.001). There were no significant RPE changes in the control condition across the different time points (all *p* > 0.05; Table 3). In the two experimental or running conditions, there were higher values of fatigue reported at mid-trial 1, mid-trial 2, 1 min post-PA, and 30 min post-PA compared to the control condition and before PA (all *p* = 0.001; Table 3). Furthermore, there were significantly higher RPE values reported in the shuttle runs condition at mid-trial 1, mid-trial 2, 1 min post-PA, and 30 min post-PA compared to the TDM and control conditions (all *p* = 0.001; Table 3).

**Table 3:** Feeling Scale (FS), Felt Arousal Scale (FAS), and rating of perceived exertion (OMNI) represented for control, shuttle runs, and TDM conditions (MEDs and IQR).

		Pre-PA	Mid-Trial 1	Mid-Trial 2	1 min Post-PA	30 min Post-PA
**FS**	Control	5 (3–5)	3 (2–5) *	3 (3–5) *	3 (3–4) *	3 (3–5)
	Shuttle runs	3 (1–5)	1 (0–3) *	−1 (−3–1) *	1 (−4–3) *	5 (3–5) *
	TDM	3 (3–5)	3 (1–3) *	3 (1–3) *	3 (1–4) *	3 (3–5)
**FAS**	Control	1 (1–2)	1 (1–1)	1 (1–1)	1 (1–2)	1 (1–2)
	Shuttle runs	1 (1–2)	5 (4–6) *	5 (5–6) *	6 (3–6) *	1 (1–2)
	TDM	2 (1–2)	3 (3–4) *	4.5 (3–6) *	4.5 (1–6) *	2 (1–3)
**OMNI**	Control	0 (0–0)	0 (0–0)	0 (0–0)	0 (0–0)	0 (0–0)
	Shuttle runs	0 (0–0)	7 (4–8) *	8 (7–9) *	9 (8–10) *	0 (0–2) *
	TDM	0 (0–0)	6 (4–6) *	6 (6–8) *	6 (4–8) *	0 (0–1) *

* Significant at *p* < 0.05 (compared to before PAs).

## 4. Discussion

This study compared response inhibition and visual and verbal recall following high-intensity intermittent shuttle runs versus The Daily Mile™, a self-paced PA bout, at 1 and 30 min post-PA in association with affect scales, rating of perceived exertion, and arousal. The results show that the shuttle runs condition improved reaction time, without a decrease in the accuracy, for the congruent stimuli at 1 min post-PA compared to the sedentary control condition.

The literature suggests that acute PA effects on EF usually tend to last after a long delay [4,7], i.e., up to 30 min, and in this study, no significant changes at 30 min post-PA following the shuttle runs and TDM were found. Although the work by Chang et al. [7] and Williams, Hatch, and Cooper [5] suggested that low–moderate-intensity PA could have immediate effects on EF performance and that these effects could persist for up to 30 min, TDM did not induce significant changes to inhibition and verbal and visual recall performance post-PA. Although there was a within-condition improvement at 1 min post-PA in the TDM condition for the congruent stimuli compared to pre-PA, this was not significant when compared to the sedentary control condition, while the shuttle runs led to improvements within and between conditions, showing a benefit over the control sedentary condition. Given this and consistent with previous studies [62], short high-intensity PA bouts seem to elicit the necessary activation, i.e., arousal, and lead to enhanced EF performance. At the same time, the null effects following the TDM condition might be explained by the lower self-reported arousal post-PA compared to the shuttle runs. The lower values of arousal reported by the participants during and after TDM might have been insufficient to induce changes in cognitive performance. Additionally, the PA dose can also be associated with the null effects observed on the visual and verbal recall tests, as the benefits in these domains are commonly reported only following ≥ 30 min duration bouts [63]. Given this, this study provides valuable information on how to modify PA opportunities in schools, as the implementation of shuttle runs in schools or modification of the actual TDM, e.g., including short bouts of high intensity, might positively impact reaction time in children post-PA.

Lambrick et al. [15] investigated the effects of continuous and intermittent PA bouts on the Stroop test and found that continuous or intermittent PA could enhance participants’ scores at 1–30 min post-PA, with larger effects for the shuttle runs condition, where both protocols were equal to 15 min of moderate intensity. In contrast, Booth et al. [19] compared the effect of a self-paced run vs. a bleep test and suggested that self-paced activity can enhance inhibition and verbal memory while the bleep test does not. High-intensity PA is usually associated with high arousal levels and is related to better EF performance post-PA [62]. Nevertheless, the timing of the assessment post-PA and the PA intensity seem to play an essential role as different time points and intensities are associated with different outcomes [7]. As Booth et al. [19] did not follow a uniform time of assessment post-PA, having only one measurement within the first 20 min post-PA and using an incremental and near to exhaustion physical test, this might have negatively impacted the results, leading to a misleading attempt of understanding the aftereffects of PA on EF. A further consideration in our study is that the EF and visual and verbal outcomes were entirely dependent on the length of the cognitive test employed; our battery of tests was 10–15 min long, with an average of 5 min for each test, and the outcomes from the different tests were given somewhere between ≈5 and 15 min post-PA. Considering that the second assessment was at 30 min post-PA, these effects might have lasted or not until the beginning of the second assessment. However, due to mental exhaustion and the requirement of having a break between cognitive tests, it is not possible to fully comprehend the extension of these improvements, and further studies are required.

Furthermore, when considering children’s academic performance, it seems more appropriate to implement school PA opportunities that can enhance physical fitness and complementarily lead to enhancements in EF performance, which are linked to academic achievement [64]. The evidence is yet too insufficient to indicate the right dose response, but it seems that short and high-intensity bouts are linked to higher levels of arousal, and this might be the mechanism responsible for these enhancements, in line with previous research in adults [62]. Moreover, when participants enjoy PA bouts, they are more likely to participate, engage, and adhere to PA [65]. In this study, when considering the Feeling Scale (FS), it seemed that participants reported lower levels of enjoyment/pleasure throughout both experimental conditions. However, when comparing the different experimental conditions at 30 min post-PA, the circumplex model showed that following the shuttle runs, significantly higher pleasure values were reported than before PA, and the displeasure felt during the shuttle runs was diminished. Thus, future research should consider ways to elicit the same intensities while creating more playful and consequently more enjoyable PA bouts to increase children’s adherence and participation.

## 5. Strengths and Limitations

The present study has several strengths, notably in comparing two different PA bouts and investigating their effects on cognitive performance, considering inhibition and verbal and visual recall, and including scales of affect before, during, and after PA bouts in school settings. Despite this, there are also some limitations. The PA protocols were only matched for duration due to the difficulty of matching these protocols for intensity in school settings and not for work or energy expenditure, creating a situation where these two protocols induced different physiological changes. Another critical factor that might have impacted our results is that TDM is a self-paced activity, and participants can perform this condition at very different paces, leading to different physiological states post-PA. Future research should consider a larger sample size and match the PA dose from both PA protocols to directly compare the qualitative type of PA, and the results in this study should be carefully interpreted. Additionally, measures of arousal in child populations might be problematic as children’s self-reported measures of arousal can differ depending on the context (i.e., pre- and post-PA), and therefore, more studies are needed to understand these variations.

## 6. Conclusions

This study shows that shuttle runs enhanced reaction time compared to the sedentary control condition, while TDM did not. However, further research is required involving different PA protocols, times of assessment, and EF tests to better understand the extent of PA’s impact on other EFs while creating more enjoyable activities to promote adherence.

## Figures and Tables

**Table 1:** Participant characteristics (Ms and SDs of distances performed and HR measures).

Variables	(Ms and SDs)	Run Variables	Control	Shuttle Runs	TDM
**Age**	9.34 (0.48)				
**Gender**	16 boys (55.2%); 13 girls (44.8%) **				
**Ethnicity ^(a)^**	22 white British (76%); 7 BAME (24%) **	**Distance**	-	837 (234.30)	1695.26 (246.14)
**Height**	140.02 (6.38)	**HR resting**	85 [10]		
**Weight**	36.34 (7.75) *	**HR before**	87 [12]	95 [8]	93 [9]
**PHV**	2.71 (1.23)	**HR during**	92 [9]	-	157 [29]
**BMI**	18.42 (2.94)	**HR active bouts**	-	*** 186 [14]	-
**550 m Run**	2.56 (0.37)	**HR rest bouts**	-	170 [10]	-
**550 m Score**	0.1 (0.99)	**HR 30 min post**	95 ± 9	110 [15]	102 [12]

Distances are represented in metres, age in years, heights in centimetres, and HR in bpm. * 31.03% overweight. ** Represented by the number of participants and percentage of the sample. ^(a)^ BAME stands for black, Asian, and minority ethnic. *** 96% of the participants achieved their predicted intensity.

## Data Availability

Data supporting the results of the current study are available on request to the corresponding author.
